# Regeneration of the Pulp Tissue: Cell Homing versus Cell Transplantation Approach: A Systematic Review

**DOI:** 10.3390/ma15238603

**Published:** 2022-12-02

**Authors:** Elisabeth Tirez, Mariano S. Pedano

**Affiliations:** Endodontics & BIOMAT—Biomaterials Research Group & UZ Leuven (University Hospitals Leuven), Dentistry, Department of Oral Health Sciences, KU Leuven (University of Leuven), 3000 Leuven, Belgium

**Keywords:** regenerative endodontics, cell homing, cell transplantation

## Abstract

Background: The main objective of this systematic review was to compare the apical healing, root maturation and histological characteristics of teeth treated with cell-based versus cell-free techniques. Methods: The methodology of this review was based on the PRISMA (Preferred Reporting Items for Systematic Reviews and Meta-Analyses) guidelines. A literature search strategy was carried out on PubMed, EMBASE and the Web of Science databases. The last search was done on 1 August 2021. Articles written in languages other than English were excluded. Two researchers independently selected the studies and extracted the data. As no randomized clinical trials were available, animal studies were included. Results: In total, 26 studies were included in the systematic review: 22 articles only researched the cell-free technique, 3 articles compared the cell-based to the cell-free technique, and 1 article compared the cell-based technique to apexification. In terms of apical healing, qualitative analysis of the data suggested that there seems to be no significant difference between cell-free and cell-based techniques. The results regarding tooth maturation are contradictory. The main difference between the cell-free and the cell-based techniques seems to be the histology of the treated tooth. The cell-free technique seems to result in cementum-like, bone-like or periodontal ligament-like tissue. One study, on the other hand, found that the cell-based technique resulted in regeneration of the whole pulp with an odontoblast layer, connective tissue, blood vessels and neuronal tissue. Conclusions: Currently, the number of randomized clinical trials on this topic are very scarce. This is probably due to the limited infrastructure and lack of resources to apply the cell-based technique. Even though both techniques seem to be promising for clinical application, long-term data need to be provided regarding the healing and reparative patterns.

## 1. Introduction

During an endodontic treatment, bacteria and infected pulp tissue are removed from the root canal of a tooth that has developed apical periodontitis due to caries, trauma or iatrogenic damage [1,2]. This is done by a combination of mechanical debridement using endodontic files and chemical irrigation using mostly sodium hypochlorite (NaOCl) and ethylenediamine tetraacetic acid (EDTA) solutions [2,3]. After the disinfection phase, the root canal is filled with a biocompatible material [4]. The main goal consists of preventing and/or eliminating apical periodontitis and resolving patient symptoms [5].

The traditional/conventional protocol for the treatment of necrotic immature teeth with open apices is apexification [6]. Apexification is a technique in which a calcium hydroxide paste or mineral trioxide aggregate (MTA) is applied to form an artificial apical barrier in the immature tooth [6]. Even though the success rates of apexification are relatively high (between 74% and 100%) [6], there are some disadvantages to this technique [6]. First, there is no continued root development of the immature tooth, and the dentin walls remain thin and thus at risk for fracture and failure [6,7]. Second, the patient needs to come back for many appointments [7,8]. Because of the long treatment period, the tooth is at risk for reinfection [9].

On the other side, regenerative endodontic treatment (RET) has become a popular research subject in endodontics over the past few decades, as it provides a promising alternative to apexification [6,10]. The concept of pulp revascularization was introduced by Dr. Nygaard-Ostby in the 1960s [6], but it was not until 2004 that a clinical protocol was introduced by Dr. Banchs and Dr. Trope [6]. The idea behind RET is to create an adequate environment to stimulate the regeneration of the neurovascular bundle (pulp tissue) of the tooth in order to restore its original function [10]; this is done by using a revitalization/revascularization protocol [10]. RET is especially useful in the treatment of immature teeth that have become necrotic (e.g., due to trauma or caries) because it is desired that continued root development occurs in these immature teeth [6]. RET provides a promising alternative to apexification because stimulation of the reduction of a periapical radiolucency and continued root development have been shown after the application of this approach in necrotic immature teeth [6].

Two main approaches have been described within RET: (1) the cell-based (cell transplantation) and (2) the cell-free (cell homing) approaches [11]. The cell-based approach relies on mesenchymal stem cell (MSC) transplantation [11]. The most commonly used MSCs for this approach are human dental pulp stem cells (hDPSCs), stem cells from the apical papilla (SCAPs) and stem cells from human exfoliated deciduous teeth (SHEDs) because of their unique properties and potential for neurogenesis and angiogenesis [10,11]. For the cell-based approach, pulp tissue is harvested (e.g., from a healthy immature tooth of the patient) and expanded in vitro [11]. The most common procedure requires collecting fresh pulp tissue from a healthy deciduous tooth of the same patient (autologous tissue) and bringing it to the lab to culture the cells. After expansion of the (stem) cells in the lab, they are brought back to the clinic to be transplanted into the disinfected necrotic tooth, together with an organic/synthetic scaffold and also combined with growth factors [11]. On the other hand, the cell-free approach relies on a process called ‘cell homing’ or ‘cell migration’ of endogenous stem cells [10]. This happens through the induction of a blood clot (e.g., by provoking bleeding with an endodontic file over the apex of the tooth) [10]. By doing this, stem cells (SCAPs or MSCs in periapical tissues) are expected to migrate to the site of injury and potentially regenerate pulp and dentin [10].

Therefore, the aim of this study was to determine whether the cell-based and the cell-free approaches result in true regeneration of the pulp/dentin complex or rather in its repair. An important note to make is that this can only be determined via tissue histology. Contrary to this, tissue histology is an aspect that is frequently missing in the literature concerning this topic. Another important aspect is the clinical relevance of true regeneration of the tooth.

The null hypothesis of this study assumes that there is no significant difference in apical healing, tooth maturation and tooth histology between the cell-based and the cell-free techniques in a permanent tooth of a young patient/animal with a periapical radiolucency and an open apex. This evaluation is performed based on an extensive literature search. Even though tooth histology is the main interest of this study, apical healing and tooth maturation were also taken into consideration because these are important clinical aspects.

## 2. Materials and Methods

### 2.1. Protocol

The methodology of this review was based on the PRISMA (Preferred Reporting Items for Systematic Reviews and Meta-Analyses) guidelines [12,13]. The protocol for this review was designed by the authors of this article.

### 2.2. Eligibility Criteria

The eligibility criteria and the inclusion/exclusion criteria for the selection of studies are shown in Table 1.

### 2.3. Information Sources

A literature search was carried out using the Pubmed, EMBASE and Web of Science databases. The first search was performed on 1 March 2021 and was updated for the last time on 1 August 2021. No filters were applied for the search. Duplicates were removed using the EndNote 20 software.

### 2.4. Search Strategy

Before performing the database search, the research question was formulated by two reviewers (MSP and ET) using the PICO structure. The chosen population was ‘young patient/animal with permanent, immature tooth with periapical infection’, the intervention was ‘cell transplantation’, the control treatment was ‘cell homing’, and the outcome was ‘regeneration’. Because randomized clinical trials were scarce on this topic, animal studies were also included. The full database searches can be found in Appendix A, Appendix B and Appendix C.

### 2.5. Study Selection and Data Collection Process

**Figure 1:**
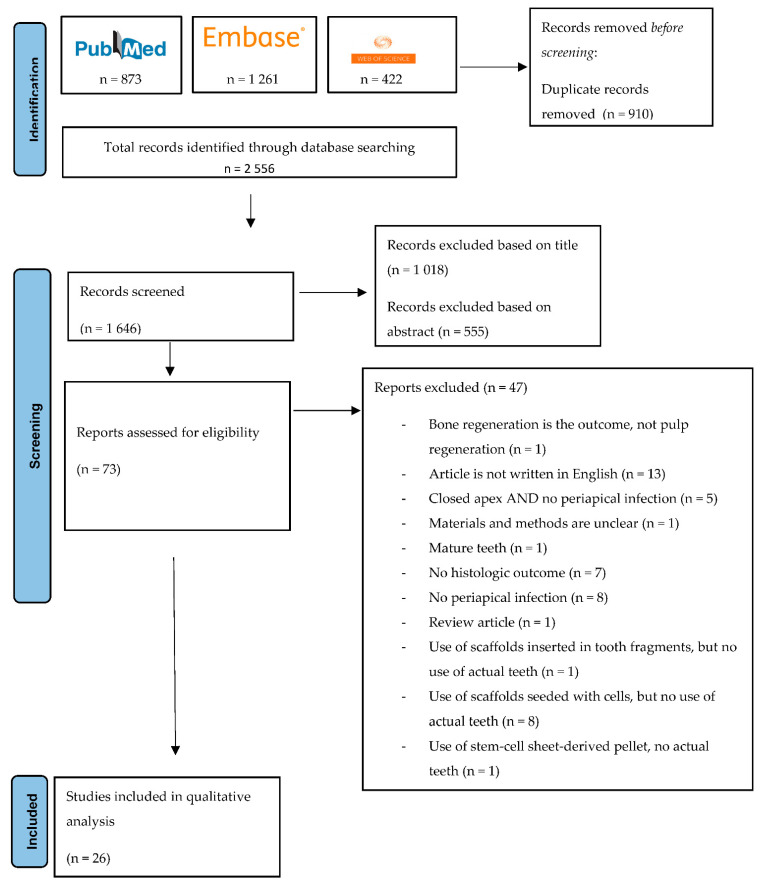
PRISMA flowchart representing the screening procedure.

Articles were screened first by title and then by abstract. Then, full-text screening was performed, and the articles that met the inclusion criteria were included. A flowchart visualising the screening procedure is shown in Figure 1.

## 3. Results Synthesis

### 3.1. Apical Healing

The studies that directly compared cell homing and cell transplantation [14,15,16] could not find a significant difference in apical healing [14,15,16]. Overall, the healing capacity seems to be sufficient in both techniques, as most of the lesions healed after treatment [14,15,16]. It seems apparent that the experimental groups (cell homing, cell transplantation) result in more apical healing than the control groups (no treatment) [16].

### 3.2. Tooth Maturation

Some studies [14,16] found that cell transplantation resulted in significantly more tooth maturation when compared to the cell-homing approach [14,16]. This in contrast to another article [15] that did not find a significant difference between the two approaches [15]. In conclusion, the results are contradictory.

### 3.3. Tissue Histology

The tissue formed after treatment with the cell homing approach generally has a cementum-like, bone-like or periodontal ligament-like structure (Table 2 and Table 3). Some studies [17,18] even described the formation of dentin-like tissue with odontoblast-like cells [17] or the formation of a hard tissue bridge [18]. One study [19] found that cell transplantation resulted in regeneration of the whole pulp with an odontoblast layer, connective tissue, blood vessels and neuronal tissue [19].

## 4. Discussion

### 4.1. Summary of Results

The results of this systematic review indicate, based on the histological analysis of the samples, that there seems to be a difference between the cell homing approach and the cell transplantation approach regarding the regeneration of the pulp. However, due to the limited number of studies available in humans and the low sample size, a clear conclusion cannot be made at this moment. Therefore, the null-hypothesis, which states that there is no significant difference in apical healing, tooth maturation and tooth histology between the cell-based (cell transplantation) and the cell-free (cell homing) techniques in a permanent tooth of a young patient/animal with a periapical radiolucency and an open apex could not be rejected.

The two regenerative endodontic treatments (RETs) that are used in regenerative endodontics today are the cell homing (cell-free) and the cell transplantation (cell-based) techniques [11]. The main interest of this study was to evaluate the difference between these two techniques in a qualitative way. Since this topic is very clinically oriented, only in vivo studies were included. However, because very few randomized/controlled clinical trials could be found on this topic, animal studies were also included. It is important to note that there might be a difference in reaction of animal pulp tissue compared to human pulp tissue [40]. This means that the results found in this systematic review, which are mainly based on animal studies, might not be completely transferable to humans.

In this review, we only included studies with patients/animals with teeth that had an open apex. One of the reasons for this is that teeth with an open apex have thin dentinal walls and are more fragile than teeth with a closed apex and thus would benefit greatly from regeneration and further maturation of the roots [6]. If the root of an immature tooth does not further develop, the tooth might be at higher risk of failure due to trauma or mechanical overload because the dentinal walls remain thin [6]. A second reason for the inclusion of teeth with open apices is that they have greater regenerative capacity compared to teeth with closed apices [10]. It has been found that a regenerated vascular network can be expected in teeth when the apical foramen is ≥0.6 mm [10]. Because the healing capacity of both techniques and the disinfection protocol was of interest, teeth with a periapical radiolucency were included.

### 4.2. Qualitative Analysis of In Vivo Studies

#### 4.2.1. Apical Healing

From analysis of the apical healing of the periapical radiolucencies of teeth treated with the cell homing technique compared to the cell transplantation technique, we may conclude that there most likely is no difference between the two techniques regarding this outcome (Table 3). The articles that directly compared the cell homing to the cell transplantation approaches [14,15,16] did not find a significant difference in apical healing between the two approaches [14,15,16]. However, it is important to note that we only found three articles [14,15,16] that directly compared the cell homing approach to the cell transplantation approach, all of which were animal studies with a limited number of subjects. It is also important to keep in mind that the majority of these results are based on radiographic findings, not histological ones.

Yamauchi et al. (2011) [36] found that the application of an insoluble cross-linked collagen scaffold resulted in significantly better apical healing compared to not using that scaffold [36]. They suggested that this phenomenon might occur due to the osseoinductive properties of the collagen scaffold [36].

An assumption has been made in the current literature that when carrying out a regenerative endodontic procedure, a higher level of disinfection needs to be attained compared to a regular root canal treatment [28]. Triple antibiotic paste is often used during the disinfection phase in a regenerative endodontic treatment (Table 2 and Table 3), and it has been proven that using a triple antibiotic paste gives better apical healing when compared to not using any intracanal medication [28]. Studies have shown that triple antibiotic paste kills bacteria inside the pulp, but that in immature teeth with open apices, bacteria possibly penetrate deeper into the dentinal tubules when compared to mature teeth with closed apices [15]. It has been shown that after regenerative treatment, immature teeth with open apices still show periapical and intracanal inflammation even though the right disinfection protocol was used (NaOCl irrigation and application of a triple antibiotic paste) [15]. More importantly, a significant association has been found between residual bacteria inside the pulp and the absence of tooth maturation [15]. Lastly, it is interesting to note that residual bacteria were always found in the coronal part of the pulp because the host response is stronger in the apical area of the tooth [15]. This means that the current challenge for the disinfection protocol used in regenerative endodontic treatments mainly lies in sufficient disinfection of the areas further away from the apex and thus the host response [15]. Recently, the concept of a ‘triple antibiotic-eluting construct’ has been proposed as a substitute for the regular triple antibiotic paste [22] because the latter has been shown to be cytotoxic [22]. More specifically, this triple antibiotic-eluting construct is a construct of antibiotic-eluting polymer nanofibers [22]. It was found that this construct provokes a less intense inflammatory reaction than the regular triple antibiotic paste [22].

Besides triple antibiotic paste, double antibiotic pastes and calcium hydroxide have also been used as a disinfection method in RET [41]. It has been found that the use of antibiotic pastes most likely results in more dentin wall thickening when compared to calcium hydroxide [41]. On the other hand, it has been suggested that the use of calcium hydroxide results in more apical closure when compared to antibiotic pastes [41]. In terms of apical healing and root lengthening, it has been found that both antibiotic pastes and calcium hydroxide deliver good results [41]. Finally, it is important to note that it has been suggested in the literature that high concentrations of antibiotics have a negative effect on apical stem cell survival, which means that the use of low concentrations of antibiotics is recommended [41].

#### 4.2.2. Tooth Maturation

From the analysis of tooth maturation of teeth treated with the cell homing technique compared to the cell transplantation technique, we may conclude that the results are contradictory regarding this outcome (Table 3). Two studies that directly compared the two techniques [14,16] found a significant difference in regards to tooth maturation [14,16]. Huang et al. (2019) [14] found that there were no significant differences between the cell homing and the cell transplantation groups in regards to root wall thickness and apical closure, but that there was a significant difference in decrease of the apical diameter [14]. More specifically, they found that the cell transplantation approach resulted in significantly more decrease of the apical diameter when compared to the cell homing approach [14]. Zhu et al. (2021) [16] found that the cell transplantation groups (using dental pulp stem cells) resulted in significantly more root wall thickening than the cell homing groups (using blood clot and platelet-rich plasma) [16]. Verma et al. (2017) [15], on the other hand, found that there was no significant difference between the two techniques regarding tooth maturation [15]. They also found that all but one of the cases without root growth showed the presence of residual bacteria [15]. Based on histobacteriologic analysis, bacteria were present in the form of thick biofilms or planktonic colonies in the part of the canal space where no tissue or necrotic tissue was present [15]. There was also a significant association between the presence of intracanal/periapical inflammation and the lack of radiographic growth [15]. The presence of bacteria was significantly associated with decreased root length, decreased apical/middle root wall thickness and the presence of intracanal inflammation [15]. Again, we should bear in mind that these results are based on animal studies with a limited number of subjects and that they are mainly based on radiographic findings instead of histological ones.

Yamauchi et al. (2011) [36] found that the application of an insoluble cross-linked collagen scaffold resulted in significantly more tooth maturation compared to not using the scaffold [36]. They suggested that this might be due to the fact that the insoluble collagen scaffold allows more time for cells to migrate and proliferate [36]. Khademi et al. (2014) [26] compared tooth maturation in vital versus necrotic teeth after using the cell homing technique and found that there was no significant difference between the two groups [26]. This implicates that using a triple antibiotic paste is effective for disinfecting the root canals during RETs [26].

Yoo et al. (2014) [17] studied the effect of revascularization using a conditioned medium from preameloblasts on tooth maturation [17]. The results of this study [17] show that revascularization using a collagen scaffold sponge soaked with conditioned medium from preameloblasts resulted in significantly more mature apices when compared to conventional revascularization using a collagen scaffold sponge washed with phosphate-buffered saline [17]. The idea behind this study [17] was that conditioned medium from preameloblasts might re-enact the microenvironment of tooth development [17].

As mentioned previously, a significant association has been found between residual bacteria inside the pulp and the absence of tooth maturation [15]. This implies that sufficient disinfection of the root canals—especially the coronal part—is of great importance for continued root development [15].

Tooth maturation as a result of the cell homing approach (cell-free approach) can be explained by the fact that if the infection in the tooth is eradicated by regenerative endodontic treatment, the function of HERS (Hertwig’s epithelial root sheath) is repaired [42]. Since HERS is responsible for the promotion of root development via the apical papilla [42], further root development of the immature tooth can be explained [42]. Another factor is that the apical papilla, which is a source of mesenchymal stem cells (MSCs), can survive apical periodontitis [42]. Tooth maturation as a result of the cell transplantation approach (cell-based approach) can be explained by the fact that this approach relies on the transplantation of human dental pulp stem cells (hDPSCs) or stem cells from human exfoliated deciduous teeth (SHED) into the pulp after seeding, culturing and expanding the cells [11]. When these exogenous cells are transplanted into the pulp with organic or synthetic scaffolds, they have potential to regenerate the dentin–pulp complex, unlike the cell homing approach [11]. This means that if the transplantation procedure is successful, de novo regeneration is achieved, which implies that the dental pulp possesses normal function and can thus induce further root formation [11].

#### 4.2.3. Tissue Histology

From the analysis of the tissue histology of teeth treated with the cell homing technique compared to the cell transplantation technique, we may conclude that there is a difference between the two techniques regarding this outcome (Table 3). This outcome is probably the most important, as tissue histology was the main interest of this study.

True regeneration or de novo regeneration implies the rehabilitation of the pulp tissue (dentin–pulp complex) and its function to the original state rather than solely revascularization [10]. In order to consider de novo regeneration of the pulp, a few key features need to be present [10]. First of all, there needs to be a new odontoblast layer lining the existing dentin [10]. The newly formed dentin usually resembles tertiary or reparative dentin instead of original dentin [10]. Secondly, a newly formed vascular and nerve network needs to be present [10]. This phenomenon seems to occur only when the apical foramen of the treated tooth is 0.6 mm [10]. This is also the reason why RETs are usually indicated when an immature tooth with an open apex has become infected or traumatised and conventional endodontic treatments (e.g., regular root canal treatment) are not possible to execute. Mature teeth with a closed apex will not respond as well to RETs because it is not likely that a newly formed vascular and nerve network will be installed, as the apical foramen has become too narrow [10]. The previously described key features can only be determined through tooth histology. This means we cannot speak of regeneration of the pulp–dentin complex when histologic proof is not present.

In general, teeth treated with the cell homing approach have mineralised tissue that is bone-like, periodontal ligament-like or cementum-like (Table 2 and Table 3). There are usually no dentin-like tissues or tubular structures to be found (Table 2 and Table 3). This means we cannot speak of true regeneration or de novo regeneration, but rather of revascularization (repair) when using the cell homing approach. However, sporadically, teeth treated with the cell homing approach do show signs of de novo regeneration. In a few cases, odontoblast-like cells are present along the dentinal wall [18,29]. In other cases, dentin-like tissue is even present [17,18,30,43].

Teeth treated with the cell transplantation approach generally have mineralized tissue that is dentin-like (Table 3). As mentioned previously, formation of dentin-like tissue is necessary in order for true regeneration or de novo regeneration to occur [10]. Only one study [19] based on human subjects was included. The analysis of this study [19] shows that cell transplantation results in regeneration of whole dental pulp tissue with an odontoblast layer, connective tissue, blood vessels and neuronal tissue [19]. It is important to note that this result is based on the histology of only one tooth. The animal studies that applied the cell transplantation technique also found dentin-like hard tissue [14,15,16]. Zhu et al. (2013) [16] also found that cell transplantation results in more mineralised tissue compared to cell homing [16].

Once again, it is important to bear in mind that the evaluation of the tissue histology was performed on animal teeth (except for one study). As mentioned previously, these results might not be completely transferable to humans [40].

The difference in tissue histology between the cell homing and the cell transplantation technique can be explained by the unique properties of hDPSCs, SCAPs and SHEDs used for the cell transplantation technique [10,11]. They have the potential for neurogenesis and angiogenesis (‘neurovascular differentiation properties’) because they have neural crest or glial origins and occupy a neurovascular niche [11]. As hDPSCs, SCAPs and SHEDs are pluripotent cells, they are capable of differentiating into pulpal cells [10,11]. The cell homing technique, on the other hand, does not use exogenous cells but relies on migration of endogenous mesenchymal stem cells (MSCs) [11]. This is a complex process, and it is not completely clear where the recruited cells come from [11]. They might come from the apical papilla, but they might also come from the circulation [11]. De novo regeneration of the pulp–dentin complex is a phenomenon that seems to be achieved only by exogenous transplantation of stem cells into the pulp [11].

Another important aspect is the clinical relevance of true regeneration. When revitalization/revascularization of the pulp–dentin occurs rather than true regeneration, apical healing and tooth maturation can still take place (Table 2). However, long-term results are needed.

### 4.3. Comparison with Previous Studies and Limitations

Recently, regenerative endodontics has become a popular topic in the literature. However, oftentimes authors do not describe the tooth histology when they speak about regeneration. As mentioned previously, this is an essential aspect. In this review, we only included articles that describe tooth histology (see Table 1) to make sure that our conclusion is as accurate as possible.

Previously, we stated in this study that there most likely is a difference between the cell homing approach and the cell transplantation approach regarding the regeneration of the pulp in a permanent tooth of a young patient/animal with a periapical radiolucency and an open apex. However, there are some limitations to this statement. All the included studies in this review are based on animals, except for one by Xuan et al. (2018) [19]. As mentioned previously, careful interpretation of these results is needed, as the reaction of animal pulp tissue might differ from the reaction of human pulp tissue [40]. This means that the results found in this systematic review might not be completely transferable to humans. Another limitation might be that only four studies were included [14,15,16,19] that applied the cell transplantation approach. This means it might be difficult to form a watertight conclusion based on so few studies. Lastly, we should note that hard-tissue histology is a difficult technique to execute and that pathologists who are experienced with tooth histology are needed in order to gather trustable information [40].

As previously mentioned, there were only four studies [14,15,16,19] included in this review that applied the cell transplantation technique. This might be because there are some practical difficulties with applying the clinical protocol for this technique, which forms a challenge for future research on this topic. First of all, there needs to be an appropriate infrastructure (good manufacturing practice facilities) in order to study the cell-based approach [10]. These are scarce because there is a high cost associated with the maintenance of such facilities as they usually use hematopoietic cell transplants [10]. A second aspect of the problem is the source of the stem cells [10]. The obtained stem cells have to be cryopreserved and stored in an appropriate way before transplantation, which again requires the correct infrastructure (e.g., stem cell banking programs) [10]. These stem cell banking programs, and, more specifically, dental stem cell banking programs, are lacking [10]. Lastly, the legal process for being able to perform a clinical trial could be an issue because stem cells that are transplanted into the host are considered drugs, which means that their use is regulated by the Food and Drug Administration or an equivalent [10]. The processing of stem cells for their use in regenerative endodontics needs to follow the standards of the (equivalent of the) Food and Drug Administration [10].

## 5. Conclusions

In conclusion, the cell-based approach seems more likely to result in true regeneration with rehabilitation of the dentin–pulp complex and the tooth’s original function than the cell-free approach (Table 2 and Table 3). However, more histological proof and clinical trials using the cell homing and the cell transplantation techniques are needed before claiming that tooth pulp regeneration has been achieved. By doing this, the clinical relevance of true regeneration will also become more evident. Moreover, care should be taken when considering these results, as most of the data come from animal studies. Currently, randomized clinical trials on this topic are very scarce. This is probably due to the limited infrastructure. Even though both techniques seem to be promising for clinical application, long-term data need to be provided.

## Figures and Tables

**Table 1:** Eligibility criteria for in vivo (human/animal) studies.

Characteristics	Inclusion Criteria	Exclusion Criteria
Language	English	Languages other than English
Ethical Committee	Mentioned	Not mentioned
Population	Human/animal permanent teeth	Human/animal deciduous teeth
	Periapical infection	No periapical infection
	Open apex (immature teeth)	Closed apex (mature teeth)
Outcome	Histologic	Only clinical/radiographic
Technique used	Cell homing/cell transplant	Only apexification
Type of study	Randomized, controlled	Other types of studies (case series, case reports, etc.)
	clinical trials (RCT or CCT)	
	or animal studies	

**Table 2:** Included articles based on cell homing approach.

Authors	No. of Subjects/Follow-Up	Groups	Disinfection Protocol between Induction of AP * and Treatment Session)	Scaffold Used	Parameters	Results
K.F. Abbas et al. (2020) [20]	8 mongrel dogs (120 premolars)/ 1 w, 1 m, 3 m	- Blood clotting (REG) - Chitosan + demineralised bone matrix (REG-CD) - Chitosan + dexamethasone corticosteroid (REG-CC) - Positive control (PC) * - Negative control (NC) *	Pulp debridement using K-files Irrigation with 20 mL 1.5% NaOCl Saline (5 min) TAP (triple antibiotic paste)	Blood clot Chitosan + demineralised bone matrix Chitosan + dexamethasone corticosteroid	Apical healing	Yes, after 3 m (except PC)
					Tooth maturation	REG, REG-CD and REG-CC > * PC after 1 m and 3 m
					Tissue histology	Coronal third: Not Specified (NS) Middle third: ~PDL *-tissue inflammatory cell infiltration and dilated blood vessels ↓ in # by time for REG, REG-CD and REG-CC Apical third: idem middle third
M. Altaii et al. (2017) [21]	4 sheep (8 incisors)/ 6 m	- Experimental (cell homing) - Control (no infection + no treatment)	Pulp debridement using K-files Irrigation with 10 mL 5.25% NaOCl Rinsing with saline and 5 mL 17% EDTA TAP	Blood clot	Apical healing	Signs of internal resorption in sheep 4
					Tooth maturation	Yes, but experimental ≈ control
					Tissue histology	Coronal third: stellate/spindle shaped cells surrounded by a mesh-like matrix with cementum- like islands and small blood vessels + hard tissue with mosaic-like matrix (≠ stages of maturation) Middle third: mature hard tissues covering the canal walls and loose fibrovascular connective tissues filling the pulp canal Apical third: similar to middle third
M.C. Bottino et al. (2019) [22]	1 beagle dog (10 premolars)/ 3 m	- Triple antibiotic-eluting constructs (TA-3Dc) - TAP (triple antibiotic paste) - Positive control - Negative control	Surface disinfection with Peridex 2% iodine tincture 10 mL 1.5% NaOCl TAP	Blood clot	Apical healing	TA-3Dc: less intense inflammatory reaction than TAP
					Tooth maturation	TA-3Dc: complete apical closure TAP: almost complete apical closure
					Tissue histology	TAP~TA-3Dc: osteodentin-like tissue (hollow hard tissue)
O. Dianat et al. (2016) [23]	3 mongrel dogs (20 teeth)/ 3 m, 6 m	- Blood clot scaffold group - PRGF * scaffold group - Positive control - Negative control	0.2% chlorhexidine (CHX) 2.5% NaOCl TAP	Blood clot (BC) PRGF	Apical healin	BC ≈ * PRGF (60% and 53.33% respectively)
					Tooth maturation	BC ≈ PRGF (60% of samples)
					Tissue histology	BC ≈ PRGF: cellular secondary cementum (apically + along root walls) + bone-like hard tissue islands
S.H. El Ashry et al. (2016) [24]	12 mongrel dogs 144 premolars)/ 2 w or 6 w or 12 w (3 equal groups)	- Blood clot - Blood clot + collagen - Blood clot + EDTA * - Blood clot + EDTA + collagen - Blood clot + MTAD * - Blood clot + MTAD + collagen - Positive control - Negative control	20 mL 2.6% NaOCl 1–2 mL TAP	Blood clot Collagen	Apical healing	After 6 w and 3 m: no significant difference between all experimental subgroups
					Tooth maturation	After 6 w and 3 m: no significant difference between all subgroups
					Tissue histology	Cementoid-like tissue (no significant difference between all subgroups after 6 w and 3 m)
H.M. El Halaby et al. (2020) [25]	9 mongrel dogs (216 roots)/ 1 m, 2 m, 3 m	- Blood clot - 17% EDTA + blood clot - Platelet-rich fibrin (PRF) - 17% EDTA + PRF - Positive control - Negative control	Povidone iodine 20 mL 1.5% NaOCl 20 mL 0.9% saline solution 1–2 mL TAP	Blood clot PRF	Apical healing	PRF: least inflammatory score amongst all experimental groups
					Tooth maturation	after 3 m: no significant difference between all experimental groups (all had (signs of) apical closure)
					Tissue histology	Osteodentin- or cementum-like or bony-like structure
A.A. Khademi et al. (2014) [26]	3 mongrel dogs (36 teeth)/3 m, 6 m	- Necrotic-infected - Vital - Positive conrol - Negative control	0.2% CHX mouthwash 20 mL 5.25% NaOCl TAP	Blood clot	Apical healing	Necrotic-infected group: 60% improvement in periapical radiolucency after 3 m
					Tooth maturation	Necrotic-infected group: 60% after 3 m Vital group: 77% after 3 m No significant difference between the 2 groups
					Tissue histology	Cementum-like, bone-like tissue
S. Moradi et al. (2016) [27]	2 dogs (28 premolars)/ 1 m, 3 m	- BC + Platelet-rich plasma (PRP) + MTA * (mineral trioxide aggregate) - BC + MTA - Positive control - Negative control	0.2% chlorhexidine 10 mL saline 10 mL 5.25% NaOCl TAP	Blood clot PRP	Apical healing	Not specified
					Tooth maturation	Newly formed vital tissues observed in 42.8% of samples in BC + PRP + MTA group and in 43.5% of samples in BC + MTA group (no significant difference between groups)
					Tissue histology	Soft connective tissues, vessels and hard mineralised tissues
C. M.L. Pagliarin et al. (2016) [28]	4 beagle dogs (40 teeth)/7 m	- TAP - 1% propolis paste - No intracanal medication - Negative control	10 mL 2.5% NaOCl Disinfection paste according to experimental group	Blood clot	Apical healing	TAP > no medication group
					Tooth maturation	Inter-group differences were not significant
					Tissue histology	Cementum-like mineralised tissue + bone-like mineralised tissue in all experimental groups
P. J Palma et al. (2017) [29]	4 beagle dogs (96 teeth)/13 w)	- Apexification - Blood Clot - Sodium Hyaluronate: chitosan scaffold (HA:CS) - Pectin:chitosan scaffold (P:CS) - Negative control - Positive control	10% iodopovidone solution 10 mL 2.5% NaOCl Ca(OH)2 OR TAP	Blood clot HA:CS P:CS	Apical healing	Apexification > BC, HA:SC and P:CS (BC: more predictable healing than HA:SC and P:CS)
					Tooth maturation	No significant difference between all experimental groups (*p* = 0.681)
					Tissue histology	- BC group: dentin formation with odontoblastlike cells in 21% of the samples - All experimental groups: homogenous mineralised tissues in the apical region
T.M.A. Saoud (2015) [30]	2 mongrel dogs (2015)/3 m	Only one group (blood clot)	5% tincture of iodine 3.5% hydrogen peroxide 5% tincture iodine 2.5% NaOCl Sterile saline solution TAP	Blood clot	Apical healing	All teeth: healed periapical lesions
					Tooth maturation	All teeth: continued root development
					Tissue histology	Mineralised tissue: bone-like and cellular cementum-like (1 case: reparative dentin-like tissue) Soft tissue: loose connective tissue, rich in blood vessels (10 cases) OR fibrous tissue, continuous with the PDL (6 cases)
S. Stambolsky et al. (2016) [31]	4 beagle dogs (40 teeth)/ 1 m, 2 m, 3 m, 4 m, 5 m, 6 m (radiographically)	NaOCl + BC (A1) NaOCl + PRP (A2) NaOCl + mTAP * + BC (B1) NaOCl + mTAP + PRP (B2) Positive control Negative control	30% hydrogen peroxide Tincture of iodine Disinfection according to experimental group (NaOCl/mTAP)	Blood clot Platelet-rich plasma	Apical healing	Not specified (NS)
					Tooth maturation	Apical closure in 34.4% of roots in experimental groups Statistical differences between all experimental groups (B2 highest percentage)
					Tissue histology	Cementum-like tissue along dentinal walls and bone-like tissue (no typical pulp tissue or new dentin)
H. Tawfik et al. (2013) [32]	9 mongrel dogs (108 teeth)/ 1 w, 3 w, 3 m	- MTA apical plug (a) - Blood clot (b) - Blood clot + injectable scaffold coated with bFGF (c) - MTA closure of empty root canal (d) - Positive control (e) - Negative control (f)	10 mL 2.6% NaOCl 1–2 mL TAP	Blood clot Scaffold coated with bFGF	Apical healing	After 3 m: a, b, c, f > d, e (*p* = 0.005) No significant differences between a, b and c
					Tooth maturation	After 3 m: b, c, f > a, e
					Tissue histology	Cementoid tissue with incremental calcifications with occasional cellular inclusions After 3 m: d and e; significantly less hard tissue scores than a, b and c
B. Thibodeau et al. (2007) [33]	6 purpose-bred mixed breed canine model dogs (60 teeth)/1 m, 2 m, 3 m	- No treatment after disinfection - Blood clot - Collagen - Collagen + blood clot - Negative control	0.12% chlorhexidine Tincture of iodine 10 mL 1.25% NaOCl TAP	Blood clot Collagen	Apical healing	64.6% of the four experimental groups showed improvement/healing of the lesions (no sig. differences between groups)
					Tooth maturation	Histological: in the 4 experimental groups; 54.9% of the roots showed apical closure (no sig. differences between groups)
					Tissue histology	No significant differences between groups (type of tissue not specified) → see article from X. Wang [18]
M. Torabinejad et al. (2015) [34]	6 ferrets (24 teeth)/3 m	- Blood clot/Gelfoam - Platelet-rich plasma (PRP) - No scaffold - Positive control	0.12% chlorhexidine 10 mL 1% NaOCl TAP	Blood clot PRP	Apical healing	PRP~blood clot/gelfoam (both 2/6 samples) No healing in the no scaffold group
					Tooth maturation	PRP or blood clot/gelfoam > no scaffold
					Tissue histology	*Coronal/middle third*: granulation tissue + inflammatory cells + dentin bridge in 1 sample of the bloodclot/gelfoam group *Apical third:* bone-like and cementum like tissue mixed with connective tissues (in 6 of 16 experimental samples)
X. Wang et al. (2010) [18]	6 purpose-bred mixed breed canine model dogs (60 teeth)/1 m, 2 m, 3 m	- No treatment after disinfection - Blood clot - Collagen - Collagen + blood clot - Negative control	0.12% chlorhexidine Tincture of iodine 10 mL 1.25% NaOCl TAP	Blood clot Collagen	Apical healing	See article B. Thibodeau et al. (2007) [33]
					Tooth maturation	See article B. Thibodeau et al. [33]
					Tissue histology	Experimental groups: cementum-like tissue (intracanal cementum), PDL-like tissue + bone-like tissue (intracanal bone like tissue) Hard tissue bridges in some samples of experimental groups One case of blood clot group: odontoblasts lining against 1 side of dentin wall
X. Xi et al. (2020) [35]	6 beagle dogs (54 teeth)/4 w, 8 w, 12 w (radiographically)	- Whole blood group - Platelet-rich plasma group (PRP) - Negative control	3% NaOCl Normal saline TAP	Blood clot PRP	Apical healing	100% of the teeth in both experimental groups
					Tooth maturation	Histological: apical closure in 65.71% of the teeth in the whole blood group and 73.33% of the teeth in the PRP group (no sig. difference between the 2 groups)
					Tissue histology	Both experimental groups: cementitious and medullary tissues in the root canals
N. Yamauchi et al. (2011) [36]	6 purpose-bred mixed breed canine model dogs (60 teeth)/1 m, 2 m, 3 m	- Blood - Blood +17% EDTA - Blood + collagen scaffold - Blood + 17% EDTA + collagen scaffold - Positive control - Negative control	0.12% chlorhexidine Tincture of iodine 10 mL 2.5% NaOCl TAP	Blood clot Cross-linked collagen scaffold (CS)	Apical healing	Groups with CS > groups without CS (*p* = 0.04)
					Tooth maturation	Groups with CS > groups without CS (*p* = 0.03)
					Tissue histology	In all treatment groups: 2 types of tissue 1 DAMT: dentin-associated mineralised tissue (few cells, without vasculature) 2 BI: bony islands (many cells, blood vessels and bone marrow- like tissues) Significantly more mineralised tissue in groups with CS
N. Yamauchi et al. (2011) [37]	6 purpose-bred mixed breed canine model dogs (60 teeth)/1 m, 2 m, 3 m	- Blood - Blood + 17% EDTA - Blood + scaffold - Blood + 17% EDTA + scaffold - Positive control - Negative control	0.12% chlorhexidine Tincture of iodine 10 mL 2.5% NaOCl TAP	Blood clot Cross-linked collagen scaffold	Apical healing	See article N. Yamauchi et al. (2011) [36]
					Tooth maturation	See article N. Yamauchi et al. (2011) [36]
					Tissue histology	DAMT: - Non-lammelar, irregular and patchy matrix - The directionality of the collagen fibers is poor and the maturity is not consistent BI: - Woven, bone-like structure - Many lacunae
Y. J. Yoo et al. (2014) [17]	3 beagle dogs (30 teeth)/1 m, 2 m, 3 m	- Conditioned medium-group (CM-group) - Conventional revascularization-group (CR-group) - Positive control - Negative control	10 mL 3.5% NaOCl TAP	Collagen scaffold Sponge Blood clot	Apical healing	76.19% for CM-group and 72.22% for CR-group (no sig. difference between groups)
					Tooth maturation	Significantly more mature apices in the CM-group
					Tissue histology	CM-group: osteodentin-like tissue (no tubule formation) Dentinlike tissue in 2 samples of CM-treated group (odontoblastlike cells)
D.D. Zhang et al. (2014) [38]	3 beagle dogs (27 teeth)/3 m	- PRP group - Blood clot group - Negative control	3% NaOCl 0.9% sterile saline TAP	PRP Blood clot	Apical healing	100% in both experimental groups
					Tooth maturation	Apical closure in 75% of PRP-group and 64.71% in BC-group (no sig. difference)
					Tissue histology	Both experimental groups: free cellular cementum-like tissue in the canal space
R. Zhou et al. (2017) [39]	3 beagle dogs (24 teeth)/3 m	- Blood clot - PRF + blood clot - Control (C)	10 mL 1.25% NaOCl	Blood clot PRF	Apical healing	100% for both experimental groups Significant difference between control and experimental groups
					Tooth maturation	BC~PRF + BC C < BC and PRF + BC
					Tissue histology	Cementocytelike cells at apex and along internal canal wall (BC~PRF + BC) Islets of bonelike tissue (BC~PRF + BC)

* AP: apical periodontitis; >: significantly greater than (*p* = 0.05); PDL: periodontal ligament; positive control: induction of infection but no treatment; negative control: no induction of infection; ≈: not significantly greater than (*p* = 0.05); PRGF: platelet-rich growth factor; MTAD: mixture tetracycline citric acid and EDTA: ethylenediamine tetraacetic acid; MTA: mineral trioxide aggegate; mTAP: modified triple antibiotic paste (ciprofloxacin, metronidazole and cefixime). #: number.

**Table 3:** Included articles based on cell transplantation approach.

Authors	No. of Subjects/Follow-Up	Groups	Disinfection Protocol between Induction of AP * and Treatment Session)	Scaffold Used	Parameters	Results
Y. Huang et al. (2019) [14]	6 beagle dogs/3 m, 6 m	- Experimental group (=transplantation) - Control group (=traditional revascularization)	20 mL 1.5 mL NaOCl 20 mL sterile saline TAP + sterile water	BC	Apical healing	No significant difference between groups (reduction of radiolucency in both groups)
					Tooth maturation	Experimental group > control group (*p* = 0.03)
					Tissue histology	Experimental group: dentin-like tissue with changes in dentinal tubule orientation Control group: cementum-like tissue
P. Verma et al. (2017) [15]	8 ferrets/3 m	- Traditional revascularization (BC) - Implanted tissue engineered construct - Positive control - Negative control	2.5% NaOCl 3 mL 1.25%NaOCl TAP	BC Hydrogen scaffold	Apical healing	All teeth without tooth maturation had residual bacteria except 1
					Tooth maturation	No significant difference between the two experimental groups
					Tissue histology	Revascularisation group: 3 teeth showed loosely organized connective tissue + mineralised tissue and 1 showed presence of osteodentin mixed with loose connective tissue Tissue engineering group: 2 teeth showed presence of osteodentin mixed with loose connective tissue Teeth without growth: bony islands in root canal
K. Xuan et al. (2018) [19]	40 humans (40 teeth)/ 1 m, 3 m, 9 m, 12 m, 24 m (safety assessment)	- Human dental pulp stem cell-group (hDPSC) - Apexification group	Not specified (‘conventional endodontic methods’)	Extracellular matrix	Apical healing	hDPSC-group after 24 m: no periapical inflammation in any of the theeth
					Tooth maturation	hDPSC-group > apexification group (*p* < 0.0001)
					Tissue histology	hDPSC group after 12 m: regeneration of whole dental pulp tissue with odontoblast layer, connective tissue, blood vessels and neuronal tissue (based on 1 tooth)
Z. Zhu et al. (2013) [16]	4 beagle dogs (32 teeth)/90 days	- Blood clot-group - Dental pulp cell-group (DPC-group) - PRP-group - DPC + PRP-group - Positive control - Negative control	10 mL 1.25% NaOCl TAP	Blood clot PRP	Apical healing	No sig. difference between experimental groups
					Tooth maturation	DPC and DPC + PRP-group > BC-group and PRP-group
					Tissue histology	New hard tissues and blood vessels in a matrix of fibrous connective tissue (PRP: more fibrous connective tissue with blood vessels) Cementum-like tissue (groups with DPCs had more more mineralized tissue formation) Bone-like tissue

## Data Availability

Not applicable.

## Appendix A. Pubmed Search

(((“Dentition, Permanent”[Mesh] OR “Permanent Dentition*”[tiab] OR “Permanent Tooth”[tiab] OR “Permanent Teeth”[tiab] OR “Permanent”[tiab] OR “Secondary Dentition*”[tiab] OR “Adult Dentition*”[tiab]) AND (((“Periapical Tissue”[Mesh] OR “Periapical Tissue*”[tiab] OR “Periapical”[tiab] OR “Apical”[tiab] OR “Apical Periodontium*”[tiab]) AND (“Infections”[Mesh] OR “Infection*”[tiab] OR “Radiolucency”[tiab] OR “Radiolucencies”[tiab] OR “Inflammation”[Mesh] OR “Inflammation*”[tiab] OR “Periodontitis”[Mesh] OR “Periodontitis”[tiab] OR “Abscess”[Mesh] OR “Abscess*”[tiab])) OR (“Infections”[Mesh] OR “Infection*”[tiab] OR “Radiolucency”[tiab] OR “Radiolucencies”[tiab] OR “Inflammation”[Mesh] OR “Inflammation*”[tiab] OR “Periapical Periodontitis”[Mesh] OR “Periapical Periodontitis”[tiab] OR “Apical Periodontitis”[tiab] OR “Periodontitis”[tiab] OR “Periodontal Abscess”[Mesh] OR “Periodontal Abscess*”[tiab] OR “Abscess*”[tiab] OR “Dental Pulp Necrosis”[Mesh] OR “Dental Pulp Necrosis”[tiab] OR “Necrosis”[tiab] OR “Necrotic”[tiab] OR “Non-Vital”[tiab])) AND ((“Tooth Apex”[Mesh] OR “Tooth Apex”[tiab] OR “Open Apical Foramen”[tiab] OR “Open Apical Foramina”[tiab] OR “Open Apex”[tiab] OR “Open Apices”[tiab]) OR (“Tooth Germ”[Mesh] OR “Tooth Germ*”[tiab] OR “Teeth Germ*”[tiab] OR “Immature tooth”[tiab] OR “Immature teeth”[tiab] OR “Immature”[tiab] OR “Growing Tooth”[tiab] OR “Growing Teeth”[tiab] OR “Tooth Maturation”[tiab] OR “Developing Tooth Root*”[tiab] OR “Developing Root*”[tiab] OR “Immature Tooth Root*”[tiab] OR “Immature Root*”[tiab] OR “Incomplete Root*”[tiab]))) OR ((“Animal Experimentation”[Mesh] OR “Animal Experimentation*”[tiab] OR “Animal Research”[tiab] OR “Animal Experimental Use*”[tiab] OR “Animal Experiment*”[tiab] OR “Animal Model*”[tiab] OR “Swine”[Mesh] OR “Swine*”[tiab] OR “Pig”[tiab] OR “Pigs”[tiab] OR “Miniature Swine*”[tiab] OR “Minipig*”[tiab] OR “Dogs”[Mesh] OR “Dog”[tiab] OR “Dogs”[tiab] OR “Rats”[Mesh] OR “Rat”[tiab] OR “Rats”[tiab] OR “Haplorhini”[Mesh] OR “Haplorhini”[tiab] OR “Monkey*”[tiab] OR “Ferrets”[Mesh] OR “Ferret*”[tiab] OR “Mustela putorius furo”[tiab] OR “Mice”[Mesh] OR “Mouse”[tiab] OR “Mice”[tiab] OR “Sheep”[Mesh] OR “Sheep”[tiab]) AND (“Tooth”[Mesh] OR “Tooth”[tiab] OR “Teeth”[tiab] OR “Dental Pulp”[Mesh] OR “Dental Pulp*”[tiab] OR “Tooth Pulp*”[tiab] OR “Teeth Pulp*”[tiab] OR “Pulp*”[tiab]))) AND (((“Stem Cell Transplantation”[Mesh] OR “Stem Cell Transplantation*”[tiab] OR “Cell Transplantation*”[tiab] OR “Cell-Based”[tiab] OR “Cell Based”[tiab]) AND (“Dental Pulp”[Mesh] OR “Dental Pulp*”[tiab] OR “Tooth Pulp*”[tiab] OR “Teeth Pulp*”[tiab] OR “Pulp*”[tiab])) OR (“Regeneration”[Mesh] OR “Regeneration*”[tiab] OR “Functional Regeneration*”[tiab] OR “De Novo Regeneration*”[tiab] OR “Regenerative Endodontics”[Mesh] OR “Regenerative Endodontics”[tiab] OR “Regenerative Medicine”[Mesh] OR “Regenerative Medicine”[tiab])) AND (“Cell Movement”[Mesh] OR “Cell Movement*”[tiab] OR “Cell Motion*”[tiab] OR “Cell Homing*”[tiab] OR “Cell Homing Approach*”[tiab] OR “Cell Migration*”[tiab] OR “Cell-Free”[tiab] OR “Cell Free”[tiab] OR “Revitalization*”[tiab] OR “Revitalisation*”[tiab] OR “Revascularisation*”[tiab] OR “Revascularization*”[tiab] OR “Non Cell Based”[tiab] OR “Non-Cell Based”[tiab] OR “Non-Cell-Based”[tiab] OR “Tooth Apex”[Mesh] OR “Tooth Apex”[tiab] OR “Apical Papilla”[tiab] OR “Blood Coagulation”[Mesh] OR “Blood Coagulation*”[tiab] OR “Blood Clot*”[tiab] OR “Blood Clotting”[tiab]) AND (“Regeneration”[Mesh] OR “Regeneration*”[tiab] OR “Pulp Regeneration*”[tiab] OR “Pulp Repair”[tiab] OR “Odontogenesis”[Mesh] OR “Odontogenesis”[tiab] OR “Tooth Growth”[tiab] OR “Teeth Growth”[tiab] OR “Tooth Development*”[tiab] OR “Teeth Development*”[tiab] OR “Root Growth”[tiab] OR “Maturation*”[tiab] OR “Tooth Maturation*”[tiab] OR “Root Elongation*”[tiab] OR “Root Development*”[tiab] OR “Histology”[Mesh] OR “Histology”[tiab] OR “Histologic*”[tiab]).

## Appendix B. Embase Search

(((‘secondary dentition’/exp OR ‘secondary dentition*’:ti,ab,kw OR ‘permanent dentition*’:ti,ab,kw OR ‘permanent tooth’:ti,ab,kw OR ‘permanent teeth’:ti,ab,kw OR ‘permanent’:ti,ab,kw OR ‘adult dentition*’:ti,ab,kw) AND (((‘periapical tissue’/exp OR ‘periapical tissue*’:ti,ab,kw OR ‘periapical’:ti,ab,kw OR ‘apical’:ti,ab,kw OR ‘apical periodontium*’:ti,ab,kw) AND (‘infection’/exp OR ‘infection*’:ti,ab,kw OR ‘radiolucency’:ti,ab,kw OR ‘radiolucencies’:ti,ab,kw OR ‘inflammation’/exp OR ‘inflammation*’:ti,ab,kw OR ‘periodontitis’/exp OR ‘periodontitis’:ti,ab,kw OR ‘abscess’/exp OR ‘abscess*’:ti,ab,kw)) OR (‘infection’/exp OR ‘infection*’:ti,ab,kw OR ‘radiolucency’:ti,ab,kw OR ‘radiolucencies’:ti,ab,kw OR ‘inflammation’/exp or ‘inflammation*’:ti,ab,kw OR ‘tooth periapical disease’/exp OR ‘tooth periapical disease*’:ti,ab,kw OR ‘periapical periodontitis’:ti,ab,kw OR ‘apical periodontitis’:ti,ab,kw OR ‘periodontitis’:ti,ab,kw OR ‘periodontal abscess’/exp OR ‘periodontal abscess*’:ti,ab,kw OR ‘abscess’:ti,ab,kw OR ‘necrosis’/exp OR ‘necrosis’:ti,ab,kw OR ‘dental pulp necrosis’:ti,ab,kw OR ‘necrotic’:ti,ab,kw OR ‘non-vital’:ti,ab,kw)) AND ((‘tooth apex’/exp OR ‘tooth apex’:ti,ab,kw OR ‘open apical foramen’:ti,ab,kw OR ‘open apical foramina’:ti,ab,kw OR ‘open apex’:ti,ab,kw OR ‘open apices’:ti,ab,kw) OR (‘tooth germ’/exp OR ‘tooth germ*’:ti,ab,kw OR ‘teeth germ*’:ti,ab,kw OR ‘immature tooth’:ti,ab,kw OR ‘immature teeth’:ti,ab,kw OR ‘immature’:ti,ab,kw OR ‘growing tooth’:ti,ab,kw OR ‘growing teeth’:ti,ab,kw OR ‘tooth maturation’:ti,ab,kw OR ‘developing tooth root*’:ti,ab,kw OR ‘developing root*’:ti,ab,kw OR ‘immature tooth root*’:ti,ab,kw OR ‘immature root*’:ti,ab,kw OR ‘incomplete root*’:ti,ab,kw))) OR ((‘animal experiment’/exp OR ‘animal experiment*’:ti,ab,kw OR ‘animal experimentation*’:ti,ab,kw OR ‘animal research’:ti,ab,kw OR ‘animal experimental use*’:ti,ab,kw OR ‘animal model*’:ti,ab,kw OR ‘pig’/exp OR ‘pig’:ti,ab,kw OR ‘pigs’:ti,ab,kw OR ‘swine*’:ti,ab,kw OR ‘miniature swine*’:ti,ab,kw OR ‘minipig*’:ti,ab,kw OR ‘dog’/exp OR ‘dog’:ti,ab,kw OR ‘dogs’:ti,ab,kw OR ‘rat’/exp OR ‘rat’:ti,ab,kw OR ‘rats’:ti,ab,kw OR ‘Haplorhini’/exp OR ‘Haplorhini’:ti,ab,kw OR ‘monkey*’:ti,ab,kw OR ‘Mustela putorius furo’/exp OR ‘Mustela putorius furo’:ti,ab,kw OR ‘ferret*’:ti,ab,kw OR ‘mouse’/exp OR ‘mouse’:ti,ab,kw OR ‘mice’:ti,ab,kw OR ‘sheep’/exp OR ‘sheep’:ti,ab,kw) AND (‘tooth’/exp OR ‘tooth’:ti,ab,kw OR ‘teeth’:ti,ab,kw OR ‘tooth pulp’/exp OR ‘tooth pulp*’:ti,ab,kw OR ‘teeth pulp*’:ti,ab,kw OR ‘dental pulp*’:ti,ab,kw OR ‘pulp*’:ti,ab,kw))) AND (((‘stem cell transplantation’/exp OR ‘stem cell transplantation*’:ti,ab,kw OR ‘cell transplantation*’:ti,ab,kw OR ‘cell-based’:ti,ab,kw OR ‘cell based’:ti,ab,kw) AND (‘tooth pulp’/exp OR ‘tooth pulp*’:ti,ab,kw OR ‘teeth pulp*’:ti,ab,kw OR ‘dental pulp*’:ti,ab,kw OR ‘pulp*’:ti,ab,kw)) OR (‘regeneration’/exp OR ‘regeneration*’:ti,ab,kw OR ‘functional regeneration*’:ti,ab,kw OR ‘de novo regeneration*’:ti,ab,kw OR ‘regenerative endodontics’/exp OR ‘regenerative endodontics’:ti,ab,kw OR ‘regenerative medicine’/exp OR ‘regenerative medicine’:ti,ab,kw)) AND (‘cell homing’/exp OR ‘cell homing*’:ti,ab,kw OR ‘cell homing approach*’:ti,ab,kw OR ‘cell motion’/exp OR ‘cell motion*’:ti,ab,kw OR ‘cell movement*’:ti,ab,kw OR ‘cell migration*’:ti,ab,kw OR ‘cell-free’:ti,ab,kw OR ‘cell free’:ti,ab,kw OR ‘revitalization*’:ti,ab,kw OR ‘revitalisation*’:ti,ab,kw OR ‘revascularization’/exp OR ‘revascularization*’:ti,ab,kw OR ‘revascularisation*’:ti,ab,kw OR ‘non cell based’:ti,ab,kw OR ‘non-cell based’:ti,ab,kw OR ‘non-cell-based’:ti,ab,kw OR ‘tooth apex’/exp OR ‘tooth apex’:ti,ab,kw OR ‘apical papilla*’:ti,ab,kw OR ‘blood clot’/exp OR ‘blood clot*’:ti,ab,kw OR ‘blood coagulation*’:ti,ab,kw OR ‘blood clotting’:ti,ab,kw) AND (‘regeneration’/exp OR ‘regeneration*’:ti,ab,kw OR ‘pulp regeneration*’:ti,ab,kw OR ‘pulp repair*’:ti,ab,kw OR ‘tooth development’/exp OR ‘tooth development*’:ti,ab,kw OR ‘teeth development*’:ti,ab,kw OR ‘odontogenesis’:ti,ab,kw OR ‘tooth growth’:ti,ab,kw OR ‘teeth growth’:ti,ab,kw OR ‘root growth’:ti,ab,kw OR ‘maturation*’:ti,ab,kw OR ‘tooth maturation*’:ti,ab,kw OR ‘root elongation*’:ti,ab,kw OR ‘root development*’:ti,ab,kw OR ‘histology’/exp OR ‘histology’:ti,ab,kw OR ‘histologic*’:ti,ab,kw).

## Appendix C. Web of Science Search

TS = ((((“secondary dentition*” OR “permanent dentition*” OR “permanent tooth” OR “permanent teeth” OR “permanent” OR “adult dentition*”) AND (((“periapical tissue*” OR “periapical” OR “apical” OR “apical periodontium*”) AND (“infection*” OR “radiolucency” OR “radiolucencies” OR “inflammation*” OR “periodontitis” OR “abscess*”)) OR (“infection*” OR “radiolucency” OR “radiolucencies” OR “inflammation*” OR “tooth periapical disease*” OR “periapical periodontitis” OR “apical periodontitis” OR “periodontitis” OR “periodontal abscess*” OR “abscess” OR “necrosis” OR “dental pulp necrosis” OR “necrotic” OR “non-vital”)) AND ((“tooth apex” OR “open apical foramen” OR “open apical foramina” OR “open apex” OR “open apices”) OR (“tooth germ*” OR “teeth germ*” OR “immature tooth” OR “immature teeth” OR “immature” OR “growing tooth” OR “growing teeth” OR “tooth maturation” OR “developing tooth root*” OR “developing root*” OR “immature tooth root*” OR “immature root*” OR “incomplete root*”))) OR ((“animal experiment*” OR “animal experimentation*” OR “animal research” OR “animal experimental use*” OR “animal model*” OR “pig” OR “pigs” OR “swine*” OR “miniature swine*” OR “minipig*” OR “dog” OR “dogs” OR “rat” OR “rats” OR “Haplorhini” OR “monkey*” OR “Mustela putorius furo” OR “ferret*” OR “mouse” OR “mice” OR “sheep”) AND (“tooth” OR “teeth” OR “tooth pulp*” OR “teeth pulp*” OR “dental pulp*” OR “pulp*”))) AND (((“stem cell transplantation*” OR “cell transplantation*” OR “cell-based” OR “cell based”) AND (“tooth pulp*” OR “teeth pulp*” OR “dental pulp*” OR “pulp*”)) OR (“regeneration*” OR “functional regeneration*” OR “de novo regeneration*” OR “regenerative endodontics” OR “regenerative medicine”)) AND (“cell homing*” OR “cell homing approach*” OR “cell motion*” OR “cell movement*” OR “cell migration*” OR “cell-free” OR “cell free” OR “revitalization*” OR “revitalisation*” OR “revascularization*” OR “revascularisation*” OR “non cell based” OR “non-cell based” OR “non-cell-based” OR “tooth apex” OR “apical papilla*” OR “blood clot*” OR “blood coagulation*” OR “blood clotting”) AND (“regeneration*” OR “pulp regeneration*” OR “pulp repair*” OR “tooth development*” OR “teeth development*” OR “odontogenesis” OR “tooth growth” OR “teeth growth” OR “root growth” OR “maturation*” OR “tooth maturation*” OR “root elongation*” OR “root development*” OR “histology” OR “histologic*”)).
